# Cross-Category Adaptation: Objects Produce Gender Adaptation in the Perception of Faces

**DOI:** 10.1371/journal.pone.0046079

**Published:** 2012-09-26

**Authors:** Amir Homayoun Javadi, Natalie Wee

**Affiliations:** 1 Section of Systems Neuroscience, Technische Universität Dresden, Dresden, Germany; 2 Institute of Cognitive Neuroscience, University College London, London, United Kingdom; 3 Cognitive Neuroscience Laboratory, Duke-NUS Graduate Medical School, Singapore, Singapore; 4 Division of Psychology and Language Sciences, University College London, London, United Kingdom; University of British Columbia, Canada

## Abstract

Adaptation aftereffects have been found for low-level visual features such as colour, motion and shape perception, as well as higher-level features such as gender, race and identity in domains such as faces and biological motion. It is not yet clear if adaptation effects in humans extend beyond this set of higher order features. The aim of this study was to investigate whether objects highly associated with one gender, e.g. high heels for females or electric shavers for males can modulate gender perception of a face. In two separate experiments, we adapted subjects to a series of objects highly associated with one gender and subsequently asked participants to judge the gender of an ambiguous face. Results showed that participants are more likely to perceive an ambiguous face as male after being exposed to objects highly associated to females and vice versa. A gender adaptation aftereffect was obtained despite the adaptor and test stimuli being from different global categories (objects and faces respectively). These findings show that our perception of gender from faces is highly affected by our environment and recent experience. This suggests two possible mechanisms: (a) that perception of the gender associated with an object shares at least some brain areas with those responsible for gender perception of faces and (b) adaptation to gender, which is a high-level concept, can modulate brain areas that are involved in facial gender perception through top-down processes.

## Introduction

If one views a white screen after prolonged exposure to a red screen, he or she will perceive it as green, red's opposite [1]. This perceptual shift in the opposite direction due to prolonged exposure to a stimulus is called ‘adaptation’. Adaptation occurs for low-level visual features such as colour [1], motion [2] and perception of shape and curvature [3].

In addition, studies have shown adaptation aftereffects to higher-order visual features in complex visual stimuli (such as faces), with dynamics of build up and decay similar to low-level visual aftereffects [4]. This has been demonstrated for attributes of faces such as race, gender [5], [6], facial expression [7], [8], perception of viewpoint [9], identity [4], [10], gaze direction [11]–[13] and the normality of distorted faces [14], [15].

Adaptation to the higher-order feature of gender has been found not only for faces, but also with other global categories of stimuli such as hands [16] and biological motion [17], [18]. Prolonged viewing of the gait of a male or female point light walker resulted in subsequent ambiguous gaits being perceived as more similar to the other gender [17]. This gender adaptation aftereffect cannot be entirely accounted for by adaptation of low-level features such as local motion: the adaptation aftereffect was significantly reduced when the points in point light walkers were dephased, suggesting the existence of neurons selective for gender.

The ability to bias gender perception of a face is also not limited to stimuli from the visual modality. It has been shown that exposure to sounds, voices and odours specific to a particular gender can also bias facial gender perception, although these are usually priming effects, meaning the preceding visual input shifts the perception of the test stimuli towards its dimension rather than away from it as in adaptation effects. Kovács et al. [19] showed that inhalation of androgen (steroid hormones that control the development and maintenance of masculine characteristics) caused men to perceive faces as more masculine as compared to when they inhaled estrogen (steroid hormones that control the development and maintenance of feminine characteristics). Similarly, Smith, Grabowecky and Suzuki [20] found that participants perceived gender-neutral faces as more masculine when presentation of the face was accompanied with pure tones in male fundamental-speaking-frequency range (the distribution of the dominant vocalization frequencies) [21] and vice versa, i.e. they perceived gender-neutral face to be more feminine when it was presented with pure tones in female fundamental-speaking-frequency range.

On a neural level, two possible mechanisms have been proposed to account for adaptation aftereffects. First, these aftereffects may be produced via bottom-up processes. For instance, adaptation may cause firing rate fatigue in neurons that selectively code for a category of a particular attribute, suppressing responses to previously experienced stimuli and allowing responses to novel stimuli to become relatively more dominant. In support of this proposal, a modelling study found that the introduction of firing-rate fatigue in simple associative neural networks was sufficient to simulate aftereffects [22]. In contrast, adaptation aftereffects may result from a top-down process. Hierarchical predictive coding models [23], [24] assert that at each stage in visual processing, bottom-up input patterns are compared against predictions which are dynamically calibrated from recent experience and the degree of prediction error is signalled. Adaptation recalibrates the top-down prediction signal, resulting in a greater prediction error, biasing percepts towards novel stimuli.

To prove that a higher-order feature is represented in the brain by a single population of neurons instead of the integration of information from distributed neural populations coding for lower-order constitutive features, one must demonstrate that adaptation to the higher-level feature cannot be accounted for by adaptation to the lower-order features that constitute it [17]. This has indeed been found in face adaptation. Although low-level aftereffects can occur following adaptation of any feature value, exposure to undistorted faces fails to produce an aftereffect [7]. In contrast, adaptation to synthetic faces which differ geometrically but do not vary in simple facial attributes is able to produce face identity aftereffects [25]. These aftereffects are invariant to changes in simple visual features of the adapting and test stimuli, including position [10], viewpoint [9], contrast, colour, size [14], and orientation [26].

Even more compelling proof that the higher-order feature is represented by a single neural population would be to demonstrate aftereffects with test stimuli from a different category as adapting stimuli. Troje et al. (2006) suggested that other classes of stimuli such as stick walkers, realistic renderings of walkers, or even faces could be tested for aftereffects following adaptation to point-light displays. As the adapting and test stimuli belong to different global categories of stimuli and thus no longer share any perceptual features, the aftereffect can only be attributed to a change in the representation of the higher-order feature common to both classes of stimuli.

The current study uses this approach to investigate whether the higher-order feature of gender is indeed coded by individual neurons tuned for gender, instead of a network of neurons coding for lower-order constitutive features as suggested by McCollough [1]. We selected objects closely associated with a particular gender as adapting stimuli and ambiguous faces that vary on a continuum of gender as test stimuli. As the adapting stimuli and the test stimuli are from different global categories of stimuli which do not share any perceptual features, any aftereffect resulting from adaptation can only be attributed to the higher-order feature of gender. To this purpose, participants viewed images of objects closely associated with a particular gender (male or female) and categorised faces (which varied from highly masculine to highly feminine) as either male or female. In line with the hypothesis that the higher-order feature of gender is coded for by single neurons, we expected to observe adaptation aftereffects, i.e. participants who view objects closely associated with males are more likely to perceive a subsequent ambiguous face as female and vice versa.

We were also interested in examining the dynamics of any adaptation aftereffects observed. It has been reported that the duration of adaptation aftereffects is highly dependent on the specific nature of the experiment. It can vary from seconds to days, e.g. up to a few seconds in low-level visual perception [27], [28] as well as in high-level visual perception [10], [29], minutes or hours [11], [30], [31] and even days [15], [32].

Three factors have been found to influence the magnitude of adaptation aftereffects measured in the test phase: (a) duration of adaptation, (b) delay between adaptation and test and (c) duration of test [15]. Rhodes et al. [33] tested the effects of adaptation duration and test duration with adaptation durations ranging from 1 s to 16 s and test durations ranging from 0.2 s to 3.2 s. They demonstrated that lasting adaptation aftereffects increase logarithmically with increasing adaptation duration and decrease exponentially with increasing test duration. Kloth and Schweinberger [11] showed that the adaptation aftereffect from adaptation to gaze direction decays over time, and can be explained by an exponential function that lasts up to 7 minutes. Furthermore, it has been shown that depending on the delay between adaptation and test experiences, two different mechanisms might be involved. Using coloured gratings as adapting stimuli, Vul, Krizay and MacLeod [34] found that magnitude of adaptation aftereffects increases with increasing duration of adaptation. They proposed that adaptation in early visual cortex happens at two distinct timescales (and perhaps within two separate systems): one that saturates and decays very quickly and another that exhibited no signs of decay within the time intervals tested.

We speculated that the nature of the task is also important in adaptation aftereffects – specifically, attention to the adapting stimuli might affect the magnitude of adaptation aftereffects. Therefore, we also explored how attention to the adapting stimuli would influence any adaptation aftereffects observed, by varying the attentional demands of the tasks related to the adapting stimuli. Two groups of participants took part in this study. Participants in the first group were asked to memorise five objects (presented for 6 s in total) and subsequently complete an old/new recognition task (experiment 1). A less demanding task was used for the second group of participants, who viewed five or six objects (presented for 6 s in total) and were required to respond by indicating the number of objects shown (experiment 2).

In order to characterise how the attentional demands of the task influenced the dynamics of the adaptation aftereffects observed, we included a five-second delay between presentation of the adapting objects and the test face in half of the trials. We expected to see (a) stronger adaptation aftereffects for the first experiment with a more engaging task compared to the second experiment and (b) any adaptation aftereffects to be highly attenuated or gone after the five-second delay.

## Methods

### Participants

A total of 30 participants (17 female, 18–24 years old) took part in this study, with half of them randomly assigned to the first experiment (memorisation task) and the rest assigned to the second experiment (counting task). Participants had normal or corrected to normal vision. They were naive to the purpose of the experiment. All participants gave written, informed consent in accordance with the Declaration of Helsinki and the guidelines approved by the University College London (UCL) ethics committee.

### Stimuli

**Figure 1:**
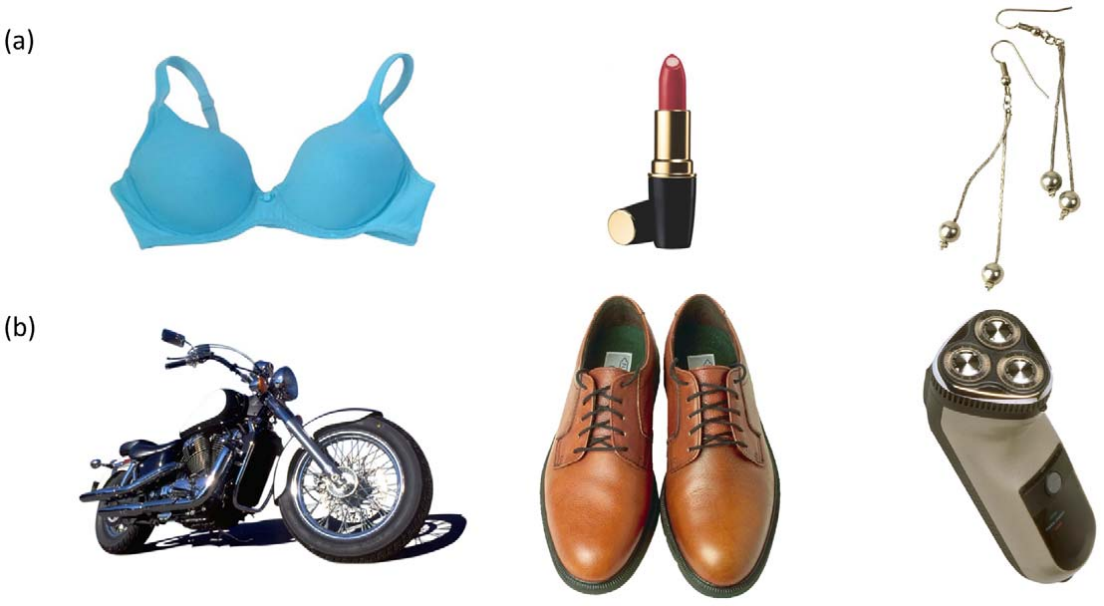
Examples of objects used for (a) female adaptation and (b) male adaptation.

Images presented in the adaptation phase comprised two sets of 49 objects that were closely associated with male and female genders, respectively. These were selected from Hemera Photo Objects (Hemera Technologies Inc.) and scaled to fit an 800×600 pixel rectangle (about 20.37×14.40 visual degrees). Examples of objects used for female and male adaptation are shown in Figure 1.

In order to ensure that these objects evoked strong associations to the intended gender, a post-study rating task was conducted in which all 98 objects were presented one by one in random order. Participants were required to categorise each object according to the gender they most closely associated it with (male or female) and respond using the keyboard by making the same key-presses as they used for the gender categorisation of faces previously (the two keys ‘J’ and ‘K’). Participants had unlimited time to make their responses. Analysis of these responses confirmed that the gender associations were indeed the ones we intended: 92.43%±2.37% of the images were similarly categorised.

**Figure 2:**
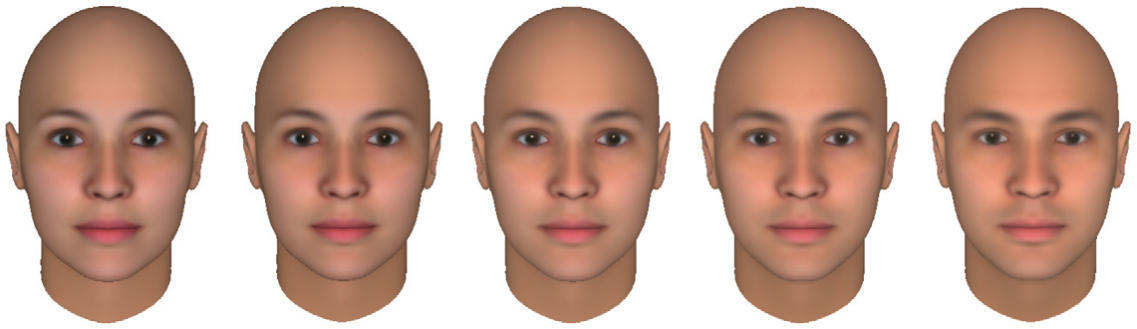
The set of test faces. From left to right: female (0% male), relatively female (20% male), neutral (50% male), relatively male (80% male) and male (100% male). In a pilot study we asked participants (n = 15) to rate the gender of 9 different faces ranging from absolutely female to absolutely male (10 repetitions per face and presented in random order) in a 5-level scale (female/somehow female/cannot guess/somehow male/male). In order to achieve an s-shape psychometric curve, we selected the faces that best fit the 0%, 20%, 50%, 80% and 100% male.

Images presented in the test phase comprised faces that were created and morphed using FaceGen Modeller (Single Inversions Inc.). All five faces (female, relatively female, neutral, relatively male and male) had a neutral emotional expression and were presented from a frontal view, Figure 2.

### Apparatus

The experiments were programmed and run with the use of MATLAB (r2007b, The MathWorks, Inc.) and Psychtoolbox v3 [35], [36]. All stimuli were presented in colour on a 17-inch monitor with a 75 Hz refresh rate and a 1280×1024 pixels resolution, at a viewing distance of approximately 53 cm. Responses were entered using a standard computer keyboard. Participants were instructed to use the index and middle fingers on their right hand to respond. The order of the keys was randomised across all participants, meaning some participants pressed the ‘J’ key to categorise faces as ‘female’ and ‘K’ as ‘male’ and vice versa for others.

### Procedure

**Figure 3:**
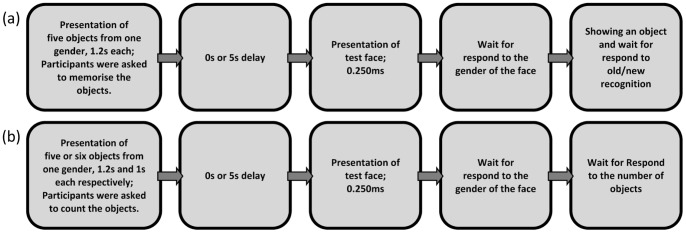
Procedure of the experiments. (a) Experiment 1 (memorisation task), (b) Experiment 2 (counting task).

Two separate experiments were conducted: the first involving a memorisation task and the second involving a less cognitively-demanding counting task. All instructions were presented on the computer screen before the experiment began. The procedure of the experiments is illustrated in Figure 3. Each trial began with an adaptation phase in which participants were adapted to a series of objects all associated with the same gender (either male or female). In both experiments, the objects were presented sequentially for 6 s. Participants were instructed to pay attention to each image as they would be tested on the objects in a later phase. Presentation of the objects was followed by either a 5 s delay (‘with-delay’ condition) or no delay (‘without-delay’ condition) before a face was presented for 250 ms. This was followed by a centrally-presented question mark on a white background, which indicated that participants were required to respond by categorising the face according to its gender.

#### Experiment 1 (memorisation task)

**Figure 4:**
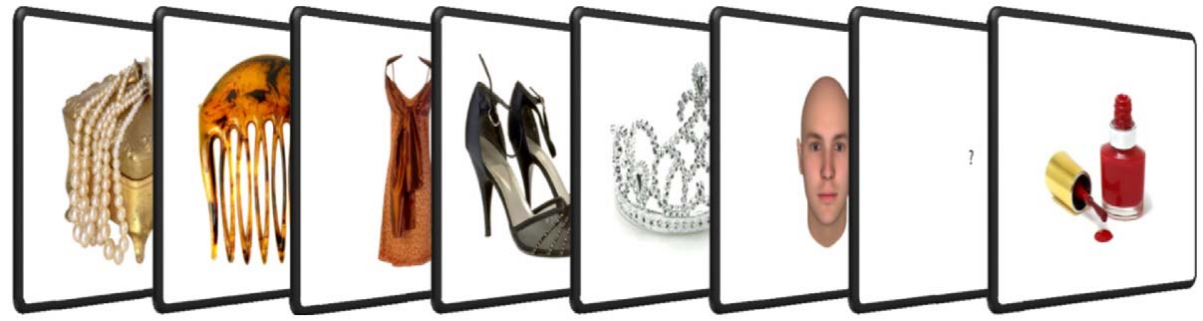
An example of a trial in experiment 1 (memorisation task). This example shows an adaptation to female-associated objects, a male face as the test stimulus with no delay between the objects and the test face and a ‘new’ object for the subsequent old/new recognition task.

Five objects were displayed in the adaptation phase for the first experiment. Participants were instructed to memorise these images for an old/new recognition task, Figure 3(a). After the gender categorisation task, an object was presented. This object could have been either presented previously in the adaptation phase (‘old’) or not (‘new’), and participants were required to respond accordingly. The procedure of a sample trial is shown in Figure 4.

#### Experiment 2 (counting task)

In the second experiment, five or six objects were displayed in the adaptation phase. Participants were asked to count the number of images, and respond if five objects or six objects were presented previously in the adaptation phase.

Each session included 12 repetitions of all the conditions (male/female objects × with-/without-delay ×5 different faces) split over 12 blocks with 20 trials in each block. Each object was presented 12 or 13 times in the first experiment and 13 or 14 times in the second experiment. Each face was presented exactly 48 times. Each block was followed by at least 30 s rest time. Participants were asked to press the ‘space’ key to continue the experiment. Participants had an unlimited time to make their responses. They did not receive any feedback on the accuracy of their responses.

### Statistical Analysis

Participants were tested on their gender categorisation judgments (male/female) of the presented faces. All analyses were carried out within-subjects using a 2×2×5 repeated-measures analysis of variance (ANOVA) in relation to three independent variables: the gender associated with the objects presented in the adaptation phase (male/female) and the delay condition (with-/without-delay) and the face types in the test phase (female/relatively female/neutral/relatively male/male). Only data from trials where participants responded correctly to the memorisation or counting task were considered for analysis. Bonferroni-corrected two-tailed paired-samples *t*-tests were used to test pair-wise comparisons post-hoc. The dependent variables were checked for normal distribution.

## Results

### Experiment 1 (memorisation task)

Table 1 summarises the results of the repeated measures ANOVA for the first experiment where participants were required to memorise the adapting stimuli. The main effects of both face type and objects' associated gender were significant (*p*<0.001) as well as their interaction (*p* = 0.02). Interactions of the delay condition with both face type and objects' associated gender were not significant (*p*>0.1). Therefore the data was collapsed across the delay conditions for post-hoc comparisons.

**Table 1:** Analysis of Variance for Experiment 1 (memorisation task).

Effect	*F*	*p*	*η_p_^2^*
delay condition	*F*(1, 14) = 0.72	0.410	ns
face type	*F*(4, 56) = 7.15	<0.001	0.61
objects' associated gender	*F*(1, 14) = 24.70	<0.001	0.71
delay condition × face type	*F*(4, 56) = 0.96	0.436	ns
delay condition × objects' associated gender	*F*(1, 14) = 0.05	0.826	ns
face type × objects' associated gender	*F*(4, 56) = 3.18	0.020	0.89
delay condition × face type × objects' associated gender	*F*(4, 56) = 1.74	0.154	ns

The results of the 2×2×5 repeated measures ANOVA with delay condition (with-/without-delay), associated gender of objects (male/female) and face type (female/relatively female/neutral/relatively male/male) as independent factors and percentage response towards male as dependent factor. *η_p_^2^* stands for partial eta-squared. *ns* stands for not significant.

**Figure 5:**
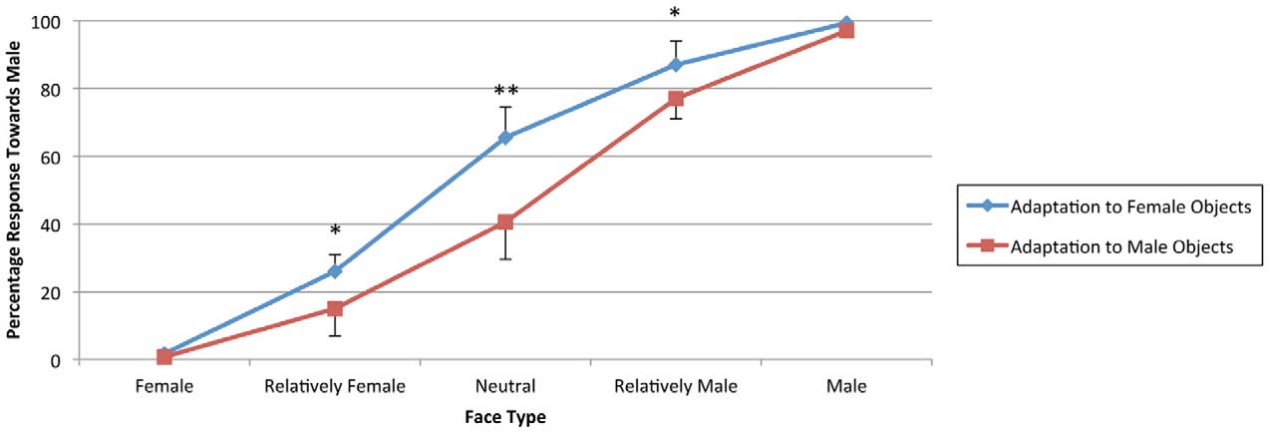
Graph of mean percentage of male responses as a function of face type in Experiment 1 (memorisation task). * *p*<0.05. ** *p*<0.01. Error bars reflect one standard deviation.

Post-hoc paired-samples *t*-tests were conducted on the percentage of responses where the face was categorized as male, in order to compare adaptation effects after exposure to objects associated with males and females for each face type, Table 2. This psychometric curve (collapsed across delay conditions) is presented in Figure 5. Critically, these tests showed a significant difference in face perception after exposure to objects associated with each gender for the relatively female (*p* = 0.03), neutral (*p* = 0.004) and relatively male (*p* = 0.01) face types. The results of the first experiment show that participants were highly adapted to the associated gender of the objects, i.e. they perceived ambiguous faces (relatively female, neutral and relatively male faces) as being more masculine after being exposed to female associated objects and vice versa. This was true regardless of the delay condition.

**Table 2:** Post-hoc Tests for Experiment 1 (memorisation task).

Face Type	*t*(14)	*p*	*d*
female	0.56	0.584	0.30
relatively female	2.41	0.030	1.29
neutral	3.41	0.004	1.82
relatively male	2.95	0.010	1.58
male	1.04	0.316	0.56

Bonferroni corrected post-hoc paired-samples *t*-tests comparing percentage response towards male for different face types after adaptation to objects associated with males and females. *d* stands for Cohen's *d* effect-size.

### Experiment 2 (counting task)

Table 3 summarises the result of the repeated measures ANOVA for the second experiment where participants were required to count the adapting stimuli. The main effect of face type (*p*<0.001) and objects' associated gender (*p* = 0.001) and their interaction (*p*<0.001) were significant. In addition, the three-way interaction between face type, objects' associated gender and the delay condition was also significant (*p* = 0.008).

**Table 3:** Analysis of Variance for Experiment 2 (counting task).

Effect	*F*	*p*	*η_p_^2^*
delay condition	*F*(1, 14) = 1.41	0.254	ns
face type	*F*(4, 56) = 6.28	<0.001	0.41
objects' associated gender	*F*(1, 14) = 17.14	0.001	0.78
delay condition × face type	*F*(4, 56) = 1.05	0.389	ns
delay condition × objects' associated gender	*F*(1, 14) = 5.18	0.039	0.58
face type × objects' associated gender	*F*(4, 56) = 7.15	<0.001	0.82
delay condition x face type × objects' associated gender	*F*(4, 56) = 3.83	0.008	0.66

The results of the 2×2×5 repeated measures ANOVA with delay condition (with-/without-delay), associated gender of objects (male/female) and face type (female/relatively female/neutral/relatively male/male) as independent factors and percentage response towards male as dependent factor. *η_p_^2^* stands for partial eta-squared. *ns* stands for not significant.

**Figure 6:**
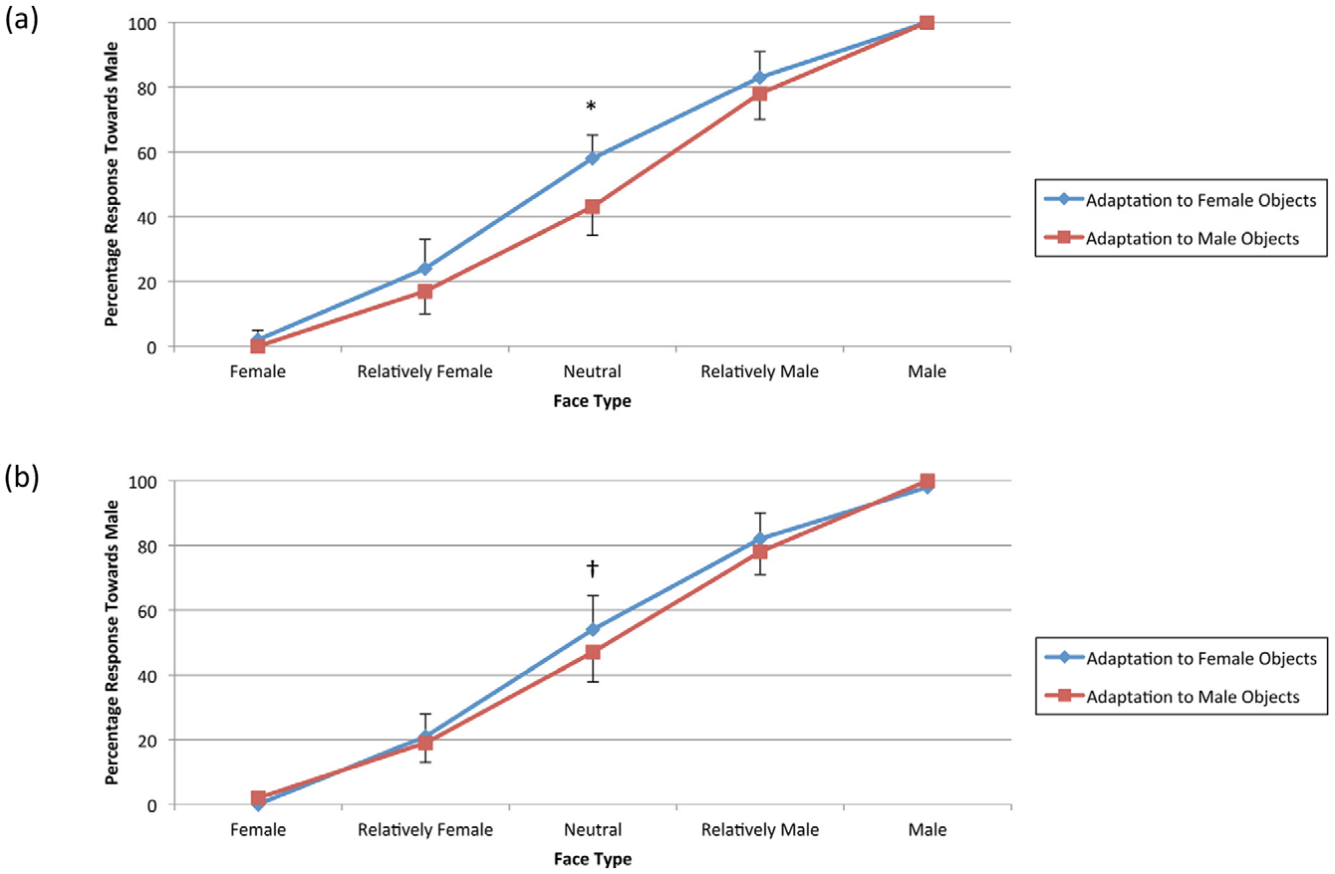
Graph of mean percentage of male responses as a function of face type in Experiment 2 (counting task). (a) without-delay condition (b) with-delay condition; * *p*<0.05, †*p* = 0.10. Error bars reflect one standard deviation.

Post-hoc paired-samples *t*-tests were conducted separately for with- and without-delay conditions to analyse the difference in response over different face types between adaptation to objects associated with males and females. Psychometric curves are presented in Figure 6 for with- and without-delay conditions. The paired-samples *t*-tests comparing the responses for the neutral face type for the without-delay condition was significant, *t*(14) = 2.57, *p* = 0.02. In contrast, the same analysis for the with-delay condition did not reach significance, *t*(14) = 1.78, *p* = 0.10. All other tests were non-significant, *ts*(14)<1. Unlike the first experiment, results of the second experiment showed differential aftereffects for the two delay conditions: participants were adapted to the associated gender of the objects when the test face was presented immediately after objects were presented (without-delay condition), but this adaptation aftereffect vanished almost entirely after 5 s of delay.

## Discussion

The current study investigated adaptation aftereffects to the higher-order feature of gender, using adapting and test stimuli from different global categories (objects and faces respectively). Specifically, we presented a series of objects highly associated with one gender with a task that required participants to attend to these adapting stimuli, and then tested participants' perception of the gender of ambiguous faces. Participants were either asked to memorise the objects presented in the adaptation phase for an old-new recognition task (Experiment 1) or to count the number of objects presented (Experiment 2), a less attentionally-demanding task. In both experiments, the test face was either presented with 5 s delay (‘with-delay’ condition) or immediately (‘without-delay’ condition) after the presentation of objects.

Results from the first experiment showed that exposure to objects highly associated with one gender caused participants to be highly biased towards the opposite gender, i.e. they were more likely to perceive the faces as male after being exposed to objects highly associated with females and vice versa, which is consistent with a gender adaptation effect (Figure 5). Contrary to our prediction, the introduction of a 5 s delay did not produce a significant difference in responses. We speculated that perhaps participants rehearsed the objects during the delay. This rehearsal might have enhanced the semantic processing of the objects, leading to a stronger adaptation aftereffect that lasted for a longer time. Alternatively, as suggested by Rhodes et al. [37], enhanced attention to the adapting stimuli may have amplified adaptation aftereffects. Rhodes et al. [37] revealed a stronger face adaptation aftereffect when participants had to pay more attention to the stimuli (using a change detection task or a 1-back task) compared to passive viewing. Therefore, to determine whether the attentional demands of the task affects the lasting effects of gender adaptation, we ran a second experiment with a less engaging task.

In the second experiment, five or six objects were presented as adapting stimuli in each trial, and participants were asked to count the number of objects. A significant two-way interaction was found between delay condition and objects' associated gender as well as a significant three-way interaction of delay condition, face type and objects' associated gender. Comparing adaptation using objects associated with males and females showed a significant adaptation aftereffect for the without-delay condition for the neutral face, but no difference for with-delay condition (Figure 6). The 5 s delay between the presentation of the objects and the test face eliminated the observed adaptation aftereffect, suggesting that this effect was temporary and attenuates greatly within 5 s.

Adaptation to gender has been previously investigated using face [16] and biological motion stimuli [17], [18]. Consistent with these studies, our findings demonstrate that perception of gender is highly influenced by past experience and subject to adaptation. However, these experiments used adapting and test stimuli which were perceptually similar as they were selected from the same global category. To our knowledge, the current study is the first to obtain a gender adaptation effect using adapting and test stimuli being from different global categories (objects and faces respectively). As the adapting and test stimuli differ perceptually, the cross-category adaptation effect observed cannot be attributed to adaptation aftereffects at the perceptual level.

Adaptation has been termed “the psychologist's microelectrode” [38] due to its power to isolate and temporarily diminish the contribution of specific neural populations, hence providing inferences about mechanisms relating to a function. The presence of adaptation is thought to imply the existence of neurons that selectively code for the adapted feature [39]. Sergent, Ohta, and McDonald [40] identified gender-related neurons in cortical areas and Kaul, Rees and Ishai [41] found a network of neurons spread over different brain areas coding gender perceived from faces. On a neural level, these higher-order features may be represented in the brain by a network of neurons, each coding selectively for the different lower-order features that constitute the higher-order feature [42], or by single neurons which selectively code for that higher-order feature [43].

Results reported in this study are consistent with Jordan et al.'s (2006) suggestion that there may be neuronal mechanisms subserving the processing of gender in the brain. This was based on the finding that the gender adaptation aftereffect was significantly reduced when adapting point light walkers were dephased, reflecting the loss of contribution of gender derived from global biological motion. However, results from the present study provide even more convincing evidence for a specific neural population coding for gender in the brain, as the adapting and test stimuli do not share any perceptual features.

The view that there exist neural populations coding for very specific concepts is supported by previous studies. For instance, opposite aftereffects were simultaneously induced in two identities in expression [8] and face-distortion experiments [14], indicating that adaptation can differentially access high-level neural populations which code for individual identities. However, this is not entirely consistent with studies that suggest that there are distinct neural populations coding for specific domains such as faces, instead of specific higher-order features such as identity and gender. Gender aftereffects were induced for hand and face categories without transfer [16], and viewpoint effects were also found not to transfer between face, car, and wire-like objects [9], indicating that separate neural populations code for each domain of stimuli. Within the domain of faces, there appear to be neural populations which are still more specific, coding for sub-domains. Opposite figural aftereffects were induced simultaneously for upright and inverted faces, indicating that distinct neural populations code for upright and inverted faces [44]. Further behavioural studies are needed to resolve this inconsistency and clarify which particular domains and/or concepts are subserved by specific neural populations.

Neuroimaging techniques, particularly the fMRI adaptation technique, may also shed light on this paradox. A recent study by Kaul et al. [41] suggests that gender information is distributed across the face network and is not specific to one area. Using multivariate pattern classification, they showed significant contributions of inferior occipital gyrus (IOG), fusiform gyrus (FG), and superior temporal sulcus (STS), as well as most of the extended regions of the face network. Future work could investigate if there is indeed a common brain area encoding gender perceived from faces and gender perceived from objects.

Extending earlier research, our results indicate that the nature of the task affects not only the magnitude of face aftereffects [37], but also their duration. We found that adaptation aftereffects lasted at least 5 s when participants were asked to ‘memorise’ the objects whereas it vanished within 5 s when participants were asked to ‘count’ the objects. Therefore, in addition to the duration of the adaptation and test phases and the delay between these two phases, the nature of the task is another crucial factor that influences the magnitude and dynamics of adaptation aftereffects.

Although participants were not informed about the aim of the study, they may have assumed a connection between the associated gender of the objects and the gender of the face. Given this assumption, it would be easier to assume that the gender of the face was congruent with the gender associated with the preceding images, rather than incongruent. The results showed that participants' perception of face gender was biased towards the gender that was not associated with the presented objects, i.e. they perceived the androgynous faces as more male after being exposed to objects associated with females and vice versa. Therefore, it is highly unlikely that the results of our study are due to the effects of social desirability.

We did not manipulate the images to control for low-level visual properties of adapting objects in order not to affect participants' perceptions of the objects. Although it remains possible that low-level visual properties contributed to our observed effects, we tried to minimise this possibility by selecting a wide variety of objects with different colours and shapes. To investigate whether there were systematic differences in colour between objects associated with males and females, we ran two independent sample *t*-tests on histogram of hue values (a value between 0 and 255 in HSV colour space) of objects in the two groups with a bin width of 16 and an overlap value of 8 (31 comparisons). These *t*-tests showed no significant difference (*p*>0.20), indicating that the low-level visual property of colour did not contribute to our observed effects.

To conclude, our findings point out that not only is our perception of gender affected by exposure to various dimensions related to humans (e.g. faces, identities, gait), but also by exposure to the objects surrounding us. The gender adaptation aftereffect arising from adaptation to objects could operate via two possible neural mechanisms – through common brain regions or through top-down processes. One possibility is that the processing of gender associated with an object shares at least some brain areas with those responsible for gender perception of faces. If this is so, such common brain areas must be highly flexible, since the association of objects with different genders are not innate, but rather learnt and may change throughout one's life. A second possibility is that perception of objects associated with a particular gender affects the activity of higher-level brain regions coding for the concept of gender, which consequently modulates the activity of lower-level visual areas, thus biasing the facial gender perception of the subsequent test face. Future studies may seek to distinguish between these two possibilities.

## References

[1] McCollough C (1965) Color adaptation of edge-detectors in the human visual system. Science 149: 1115–1116. 17737844 10.1126/science.149.3688.1115

[2] Huk A, Ress D, Heeger D (2001) Neuronal basis of the motion aftereffect reconsidered. Neuron 32: 161–172. 11604147 10.1016/s0896-6273(01)00452-4

[3] Gibson J (1933) Adaptation, after-effect and contrast in the perception of curved lines. Journal of Experimental Psychology 16: 1–31.

[4] Leopold D, Rhodes G, Müller K, Jeffery L (2005) The dynamics of visual adaptation to faces. Proceedings of the Royal Society B: Biological Sciences 272: 897–904. 16024343 10.1098/rspb.2004.3022 PMC1564098

[5] Webster M, Kaping D, Mizokami Y, Duhamel P (2004) Adaptation to natural facial categories. Nature 428: 557–561. 15058304 10.1038/nature02420

[6] Kloth N, Schweinberger SR, Kovács G (2010) Neural correlates of generic versus gender-specific face adaptation. Journal of Cognitive Neuroscience 22: 2345–2356. 19702459 10.1162/jocn.2009.21329

[7] Webster M, MacLin O (1999) Figural aftereffects in the perception of faces. Psychonomic Bulletin & Review 6: 647–653. 10682208 10.3758/bf03212974

[8] Hsu S, Young A (2004) Adaptation effects in facial expression recognition. Visual Cognition 11: 871–899.

[9] Fang F, He S (2005) Viewer-centered object representation in the human visual system revealed by viewpoint aftereffects. Neuron 45: 793–800. 15748853 10.1016/j.neuron.2005.01.037

[10] Leopold D, O'Toole A, Vetter T, Blanz V, Wild H, et al (2001) Prototype-referenced shape encoding revealed by high-level after effects. Nature Neuroscience 4: 89–94. 11135650 10.1038/82947

[11] Kloth N, Schweinberger SR (2008) The temporal decay of eye gaze adaptation effects. Journal of Vision 8. 10.1167/8.11.4 18831598

[12] Seyama J, Nagayama RS (2006) Eye direction aftereffect. Psychological research 70: 59–67. 15378364 10.1007/s00426-004-0188-3

[13] Jenkins R, Beaver JD, Calder AJ (2006) I Thought You Were Looking at Me Direction-Specific Aftereffects in Gaze Perception. Psychological Science 17: 506–513. 16771801 10.1111/j.1467-9280.2006.01736.x

[14] Yamashita J, Hardy J, De Valois K, Webster M (2005) Stimulus selectivity of figural aftereffects for faces. Journal of Experimental Psychology: Human Perception and Performance 31: 420–437. 15982123 10.1037/0096-1523.31.3.420

[15] Carbon CC, Ditye T (2011) Sustained effects of adaptation on the perception of familiar faces. Journal of Experimental Psychology: Human Perception and Performance 37: 615–625. 20731521 10.1037/a0019949

[16] Kovács G, Zimmer M, Banko E, Harza I, Antal A, et al (2006) Electrophysiological correlates of visual adaptation to faces and body parts in humans. Cerebral Cortex 16: 742–753. 16120795 10.1093/cercor/bhj020

[17] Jordan H, Fallah M, Stoner G (2006) Adaptation of gender derived from biological motion. Nature Neuroscience 9: 738–739. 16715080 10.1038/nn1710

[18] Troje N, Sadr J, Geyer H, Nakayama K (2006) Adaptation aftereffects in the perception of gender from biological motion. Journal of Vision 6: 850–857. 16895463 10.1167/6.8.7

[19] Kovács G, Gulyás B, Savic I, Perrett DI, Cornwell RE, et al (2004) Smelling human sex hormone-like compounds affects face gender judgment of men. Neuroreport 15: 1275–1277. 15167548 10.1097/01.wnr.0000130234.51411.0e

[20] Smith EL, Grabowecky M, Suzuki S (2007) Auditory-visual crossmodal integration in perception of face gender. Current Biology 17: 1680–1685. 17825561 10.1016/j.cub.2007.08.043

[21] Deliyski D, Gress CD. Characteristics of motor speech performance: normative data; 1997; Boston, MA. 3600.

[22] Menghini F, van Rijsbergen N, Treves A (2007) Modelling adaptation aftereffects in associative memory. Neurocomputing 70: 2000–2004.

[23] Friston K (2005) A theory of cortical responses. Philosophical Transactions of the Royal Society B: Biological Sciences 360: 815–836. 10.1098/rstb.2005.1622 PMC1569488 15937014

[24] Rao R, Ballard D (1999) Predictive coding in the visual cortex: a functional interpretation of some extra-classical receptive-field effects. Nature Neuroscience 2: 79–87. 10195184 10.1038/4580

[25] Anderson ND, Wilson HR (2005) The nature of synthetic face adaptation. Vision Research 45: 1815–1828. 15797771 10.1016/j.visres.2005.01.012

[26] Watson T, Clifford C (2003) Pulling faces: An investigation of the face-distortion aftereffect. Perception – London 32: 1109–1116. 14651323 10.1068/p5082

[27] Bales J, Follansbee G (1935) The after-effect of the perception of curved lines. Journal of Experimental Psychology 18: 499–503.

[28] Hammer ER (1949) Temporal factors in figural after-effects. The American Journal of Psychology 62: 337–354. 18134357

[29] Hurlbert A (2001) Trading faces. Nature Neuroscience 4: 3–4. 11135632 10.1038/82877

[30] Carbon CC, Strobach T, Langton SRH, Harsányi G, Leder H, et al (2007) Adaptation effects of highly familiar faces: Immediate and long lasting. Memory & Cognition 35: 1966–1976. 18265612 10.3758/bf03192929

[31] McKone E, Edwards M, Robbins R, Anderson R (2005) The stickiness of face adaptation aftereffects. Journal of Vision 5: 822–822.

[32] Neitz J, Carroll J, Yamauchi Y, Neitz M, Williams DR (2002) Color perception is mediated by a plastic neural mechanism that is adjustable in adults. Neuron 35: 783–792. 12194876 10.1016/s0896-6273(02)00818-8

[33] Rhodes G, Jeffery L, Clifford CWG, Leopold DA (2007) The timecourse of higher-level face aftereffects. Vision Research 47: 2291–2296. 17619045 10.1016/j.visres.2007.05.012

[34] Vul E, Krizay E, MacLeod DIA (2008) The McCollough effect reflects permanent and transient adaptation in early visual cortex. Journal of Vision 8. 10.1167/8.12.4 18831617

[35] Brainard D (1997) The psychophysics toolbox. Spatial vision 10: 433–436. 9176952

[36] Pelli D (1997) The VideoToolbox software for visual psychophysics: Transforming numbers into movies. Spatial vision 10: 437–442. 9176953

[37] Rhodes G, Jeffery L, Evangelista E, Ewing L, Peters M, et al (2011) Enhanced attention amplifies face adaptation. Vision Research 51: 1811–1819. 21704059 10.1016/j.visres.2011.06.008

[38] Frisby J (1979) Seeing: Illusion, brain, and mind: Oxford University Press.

[39] Blakemore C, Campbell F (1969) On the existence of neurones in the human visual system selectively sensitive to the orientation and size of retinal images. The Journal of physiology 203: 237–260. 5821879 10.1113/jphysiol.1969.sp008862 PMC1351526

[40] Sergent J, Ohta S, McDonald B (1992) Functional neuroanatomy of face and object processing: a positron emission tomography study. Brain 115: 1115–1116. 10.1093/brain/115.1.15 1559150

[41] Kaul C, Rees G, Ishai A (2011) The Gender of Face Stimuli is Represented in Multiple Regions in the Human Brain. Frontiers in Human Neuroscience 4. 10.3389/fnhum.2010.00238 PMC3026581 21270947

[42] Gross C, Bender D, Rocha-Miranda C (1969) Visual receptive fields of neurons in inferotemporal cortex of the monkey. Science 166: 1303–1306. 4982685 10.1126/science.166.3910.1303

[43] Kanwisher N, McDermott J, Chun MM (1997) The fusiform face area: a module in human extrastriate cortex specialized for face perception. The Journal of Neuroscience 17: 4302–4311. 9151747 10.1523/JNEUROSCI.17-11-04302.1997 PMC6573547

[44] Rhodes G, Jeffery L, Watson TL, Jaquet E, Winkler C, et al (2004) Orientation-contingent face aftereffects and implications for face-coding mechanisms. Current Biology 14: 2119–2123. 15589154 10.1016/j.cub.2004.11.053

